# Controlled reduction for size selective synthesis of thiolate-protected gold nanoclusters Au_n_(n = 20, 24, 39, 40)

**DOI:** 10.1186/1556-276X-7-277

**Published:** 2012-05-30

**Authors:** Xiangming Meng, Zhao Liu, Manzhou Zhu, Rongchao Jin

**Affiliations:** 1 Department of Chemistry, Anhui University, Hefei, Anhui, 230026, People’s Republic of China; 2 Department of Chemistry, Carnegie Mellon University, Pittsburgh, PA, 15213, USA

**Keywords:** Gold nanoclusters, Size selective synthesis, Controlled reduction

## Abstract

This work presents a controlled reduction method for the selective synthesis of different sized gold nanoclusters protected by thiolate (SR = SC_2_H_4_Ph). Starting with Au(III) salt, all the syntheses of Au_*n*_(SR)_*m*_ nanoclusters with (*n*, *m*) = (20, 16), (24, 20), (39, 29), and (40, 30) necessitate experimental conditions of slow stirring and slow reduction of Au(I) intermediate species. By controlling the reaction kinetics for the reduction of Au(I) into clusters by NaBH_4_, different sized gold nanoclusters are selectively obtained. Two factors are identified to be important for the selective growth of Au_20_, Au_24_, and Au_39/40_ nanoclusters, including the stirring speed of the Au(I) solution and the NaBH_4_ addition speed during the step of Au(I) reduction to clusters. When comparing with the synthesis of Au_25_(SC_2_H_4_Ph)_18_ nanoclusters, we further identified that the reduction degree of Au(I) by NaBH_4_ also plays an important role in controlling cluster size. Overall, our results demonstrate the feasibility of attaining new sizes of gold nanoclusters via a controlled reduction route.

## Background

Gold nanoclusters [1-6] have received extensive attention owing to their interesting optical properties [6-9], magnetism [10,11], fluorescence [12-16], chirality [17-20], redox properties [21-27], as well as potential applications in many fields such as catalysis and biological labeling [28-33]. The new physicochemical properties of gold nanoclusters are largely imparted by the discrete electronic structure of the metal core due to quantum confinement effects. The surface of the cluster may also influence some of the material properties, such as chirality [18,19].

Recently, major advances in wet chemical synthesis of nanoclusters have been achieved, and it has been possible to control nanoclusters at the atomic level. A number of well-defined nanoclusters have been reported; however, only a few can be obtained in bulk quantities and in high yields via facile synthetic methods [34]. Among the various thiolate-protected gold nanoclusters, Au_25_(SR)_18_ has been extensively studied [21-27,35-41]. Other well-defined nanoclusters have also been attained, such as Au_36_[42], Au_38_[43,44], Au_102_[45], and Au_144_[46,47].

We previously reported a kinetically controlled synthetic approach for the synthesis of highly pure Au_25_(SR)_18_ nanoclusters [48,49]. The method involves a size focusing mechanism, that is, the initial cluster product of mixed sizes is converged to a specific size of highest stability under appropriate conditions [34]. By controlling the size range of the initial nanoclusters, one can achieve atomic monidispersity of nanoclusters [34]. This synthetic approach constitutes a versatile strategy for gold nanocluster synthesis [49] and has been demonstrated in the synthesis of quite a number of atomically precise Au_*n*_(SR)_*m*_ nanoclusters, such as Au_25_, Au_38_, and Au_144_[34].

Herein, we demonstrate that a controlled reduction method can lead to different sizes of gold nanoclusters. By making a modification of the synthetic method of Au_24_ nanoclusters [50], we have obtained two new sizes, including Au_39_(SC_2_H_4_Ph)_29_ and Au_40_(SC_2_H_4_Ph)_30_. Our results explicitly show that the initial growth stage of nanoclusters is critical and can be largely influenced by experimental conditions. This method of controlled reduction has expanded the synthetic approaches for preparing nanoclusters with size control.

## Methods

### Materials

The following chemicals were used: tetrachloroauric(III) acid (HAuCl_4_·3H_2_O, >99.99 % metals basis, Sigma-Aldrich Corporation, St. Louis, MO, USA), tetraoctylammonium bromide (TOAB, ≥98%, Fluka Chemicals Limited, Gillingham, Medway, UK), phenylethanethiol (PhC_2_H_4_SH, 99%, Acros Organics, Thermo Fisher Scientific, NJ, USA), and sodium borohydride (99.99%, metals basis, Sigma-Aldrich). The solvents include toluene (HPLC grade, ≥99.9%, Sigma-Aldrich), ethanol (absolute, 200 proof, PHARMCO-AAPER, Shelbyville, KY, USA). Pure water was from Wahaha Co. LTD (Hangzhou, China). All glassware was thoroughly cleaned with *aqua regia* (HCl: HNO_3_ = 3:1 vol), rinsed with copious pure water, and then dried in an oven prior to use.

### Analysis tools

All UV-visible (vis) absorption spectra of Au nanoclusters in either toluene or methylene chloride were recorded using a Hewlett-Packard (HP, Palo Alto, CA, USA) 8453 diode array spectrophotometer. Electrospray ionization mass spectra were acquired using a Waters Q-TOF (Waters Corporation, Milford, MA, USA) mass spectrometer equipped with a Z-spray source. The sample solution (approximately 1 mg/mL) dissolved in toluene was diluted in dry methanol (50 mM cesium acetate CsAc, 1:2 vol). The sample was directly infused at 5 μL/min. The source temperature was kept at 70°C. The spray voltage was kept at 2.20 kV; the cone voltage, at 60 V.

### Synthesis of Au_*n*_ nanoclusters (*n* = 39 and 40)

HAuCl_4_·3H_2_O (0.1612 g, 0.41 mmol) was dissolved in 5 mL water, and tetraoctylammonium bromide (TOAB, 0.2541 g, 0.465 mmol) was dissolved in 10 mL toluene. These two solutions were combined in a 25-mL tri-neck, round-bottom flask. The solution was vigorously stirred (approximately 1,100 rpm) with a magnetic stir bar to facilitate phase transfer of Au(III) salt into the toluene phase. After approximately 15 min, phase transfer was completed; the clear aqueous phase was then removed. The toluene solution was cooled down to 0 °C in an ice bath over a period of approximately 30 min under constant magnetic stirring. After that, magnetic stirring was reduced to a slow speed (approximately 100 rpm), PhC_2_H_4_SH (0.20 mL, approximately threefold the moles of gold) was added, and the solution was kept under slow stirring. The solution color changed slowly from deep red to faint yellow and to colorless over approximately 1 h. After that, the speed of magnetic stirring was increased from approximately 100 to 400 rpm. At the same time, 1 mL aqueous solution of NaBH_4_ (0.44 mol/L, freshly made with ice-cold water) was dropwise added to the toluene solution over a 15-min period using a 1-mL syringe. The color of the solution turned black gradually. After the dropwise addition of NaBH_4_, the reaction was allowed to further proceed overnight. The optical absorption spectrum of the crude reaction product (diluted with toluene) shows a distinct absorption band at approximately 800 nm.

### Post-synthetic treatment of the crude product

The aqueous layer at the bottom of the flask was removed using a syringe, and the toluene solution was concentrated by rotary evaporation at room temperature. Ethanol (approximately 50 mL) was added to precipitate the Au nanoclusters. The brown, turbid solution was allowed to stand on bench for several hours. The precipitate was collected and redissolved in toluene. This precipitation/dissolution process was repeated with ethanol. The crude mixture was extracted with methylene chloride/acetonitrile (1:9 vol) to remove a small amount of Au_20_(SC_2_H_4_Ph)_16_ (its optical absorption band at approximately 485 nm) [51]. After Au_20_ was removed from the product, Au_24_(SC_2_H_4_Ph)_20_ nanoclusters were removed by a second extraction with methylene chloride/acetonitrile (1:2 vol) [50]. The final remaining product was collected and characterized by mass spectrometry.

## Results and discussion

### Identification of Au_39_(SC_2_H_4_Ph)_29_ and Au_40_(SC_2_H_4_Ph)_30_

Starting with an Au(III) salt precursor, the synthesis of gold nanoclusters involves two primary stages: (a) reduction of Au(III) to Au(I) by HSR, during which the formed Au(I) intermediate species spontaneously aggregates into polymeric Au(I) species (unknown structure), and (b) reduction of Au(I) to Au_*n*_(SR)_*m*_ nanoclusters by NaBH_4_.

In this work, we have identified several important factors for the synthesis of nanoclusters Au_39_ and Au_40_, including the stirring speed of the reaction mixture, the addition speed, and the amount of NaBH_4_ solution to reduce Au(I) into clusters. The synthetic conditions reported in this work differ from the previous syntheses of Au_19_(SC_2_H_4_Ph)_13_, Au_20_(SC_2_H_4_Ph)_16_ and Au_24_(SC_2_H_4_Ph)_20_ (see ‘Methods’ section) [50-52]. Specifically, in the present work, our major modification lies in the *stirring speed* of the Au(I) intermediate solution when reduced by NaBH_4_. In a previous work, Au_20_(SC_2_H_4_Ph)_16_ and Au_24_(SC_2_H_4_Ph)_20_ were synthesized by controlling the stirring speed for the reduction step of Au(I) by NaBH_4_; for example, approximately 50 rpm for Au_20_(SC_2_H_4_Ph)_16_ and approximately 100 rpm for Au_24_(SC_2_H_4_Ph)_20_.

**Figure 1:**
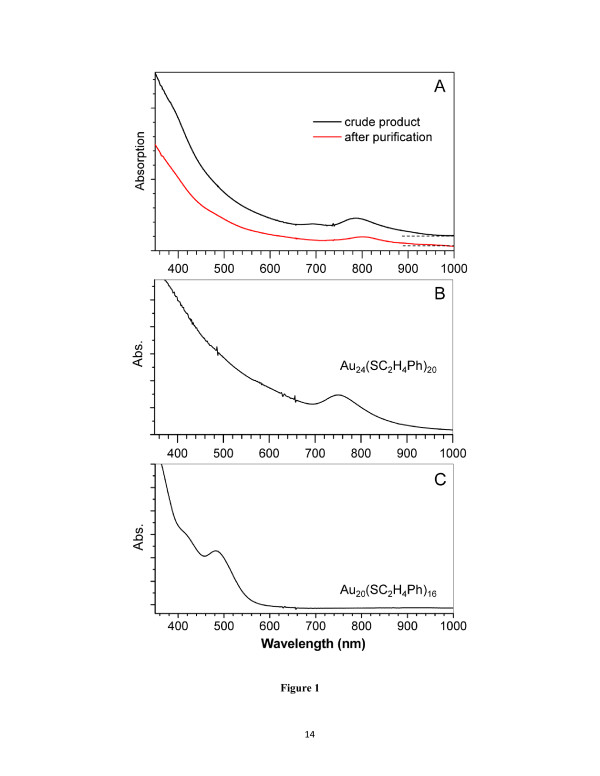
**UV-vis absorption spectra.** (**A**) The new cluster product (black profile, crude product without any extraction; red, after two extractions), (**B**) pure Au_24_(SC_2_H_4_Ph)_20_, and (**C**) pure Au_20_(SC_2_H_4_Ph)_16._

To synthesize larger-sized nanoclusters, we rationalize that the kinetics of the reduction reaction of Au(I) intermediate species by NaBH_4_ may be important for potential size control. Motivated by that, we systematically varied the synthetic conditions and also compared with the typical method for Au_25_(SC_2_H_4_Ph)_18_ synthesis. Interestingly, we found that with the stirring speed being increased to approximately 400 rpm, the crude product (Figure 1A) shows an optical spectrum different from that of Au_24_(SC_2_H_4_Ph)_20_ or Au_20_(SC_2_H_4_Ph)_16_ (Figure 1B,C). A new absorption peak centered at approximately 800 nm was observed (Figure 1A), indicating that some new species have been formed in this controlled reduction process. Of note, the small peak at approximately 700 nm (Figure 1A) is due to the concurrent formation of a small amount of Au_24_(SC_2_H_4_Ph)_20_ clusters as impurities in the synthesis of the new clusters. To remove Au_24_ and possible Au_20_ impurities from the product, the clusters in the crude product were precipitated by adding ethanol, and the crude product was then extracted with CH_2_Cl_2_/CH_3_CN (1:9 vol) to selectively dissolve Au_20_(SC_2_H_4_Ph)_16_ from the product. The remained undissolved produce was followed by a second extraction with CH_2_Cl_2_/CH_3_CN (1:2 vol) to remove Au_24_(SC_2_H_4_Ph)_20_. The final remaining product is largely free of Au_24_ and Au_20_ impurities, as evidenced in the disappearance of the 700-nm band in the optical spectrum (Figure 1A, red profile). The relatively pure product is subject to further characterization for cluster formula determination.

We employ electrospray ionization mass spectrometry (ESI-MS) to determine the composition of the new gold cluster product. A solution of cesium acetate (CsAc, 50 mM, in dry methanol) was added to a toluene solution of gold clusters at 1:1 or 1:2 (vol). ESI-MS detects the cluster-Cs adducts that are positively charged due to Cs^+^ addition to the cluster surface.

**Figure 2:**
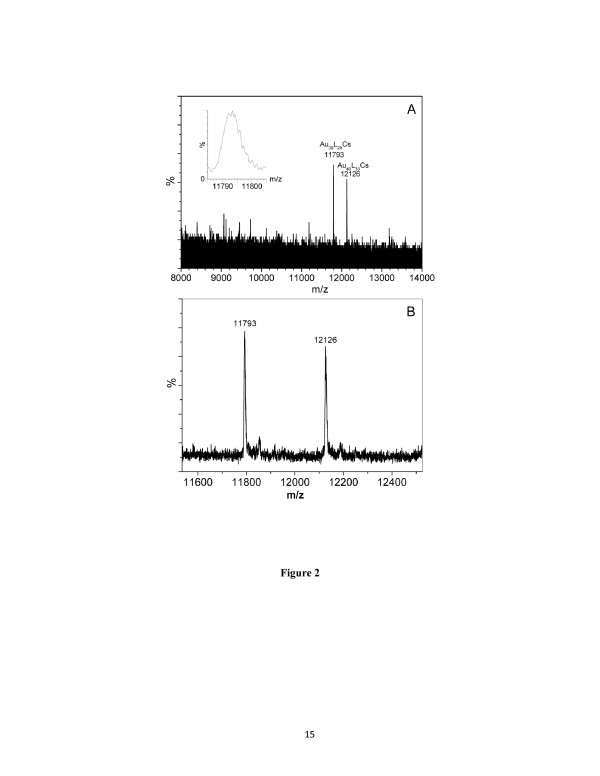
**ESI-MS spectra of the new nanolcusters.** (**A **) Unsmoothed spectrum in the range of *m*/*z* 8,000 to 14,000 (inset, isotope pattern of *m*/*z* 11,793) and (**B**) zoom-in spectrum (smoothed).

The low-mass portion of the spectrum consists of all [CsAc]_*x*_Cs^+^ signals (Figure S1 in Additional file 1), and residual Au_24_(SC_2_H_4_Ph)_20_Cs was also observed at *m*/*z* 7,603 (calculated FW = 7604 for mono-Cs adduct). The high-mass portion (Figure 2) shows two peaks at *m*/*z* 11,793 and 12,127 (Figure 2A, unsmoothed spectrum; Figure 2B, smoothed). These two signals are corresponding to the new clusters formed in the controlled reduction process. Both peaks indicate +1 ions, evidenced by the unity spacing of the isotope-resolved peaks (see Figure 2A inset, for *m*/*z* 11,793). After subtracting one Cs^+^ ion (FW = 133), the masses of the two new clusters are determined to be 11,660 and 11,994 Da. Unfortunately, isolation of these two mixed clusters into pure ones has not been successful so far.

To deduce the formulas of the two new Au_*n*_(SC_2_H_4_Ph)_*m*_ clusters based upon the ESI-MS data, we first determine the minimum value of the gold atom number (*n*), which corresponds to the limiting case of Au(I):SR complexes, i.e., when *n* = *m*. This gives rise to 11,660/337 approximately 35 for the peak of *m*/*z* 11,660. We then construct candidate formulas by systematically increasing *n* starting from 35. For example, for *n* = 36, we list two closest matches to the ESI-determined mass of 11,660 Da, one with the smallest negative deviation, i.e., (*n*, *m*) = (36, 33), and the other with the smallest positive deviation, i.e., (*n*, *m*) = (36, 34), see Table 1; note that other *m* values for *n* = 36 are not pursued since they deviate more from the ESI mass of 11,660 Da. Following this method, one can list all the possible formulas until the limiting case of 11,660 Da consisting of all gold atoms, which is the upper limiting case since a certain number of thiolate ligands must be present to protect the metal core. By comparing the candidate formulas with the ESI-MS-determined precise mass of 11,660 Da, we readily obtain the Au_39_(SC_2_H_4_Ph)_29_ formula (calculated FW = 11661; deviation, 1 Da), as reflected in Table 1. Similarly, the cluster of 11,993 Da is determined to be Au_40_(SC_2_H_4_Ph)_30_ (calculated FW = 11,995; deviation, 2 Da). A deviation of 0.5 to 2 Da is reasonable at this high mass range.

**Table 1:** List of candidate formulas for the two clusters

**(*n*,*m*) Candidate formulas for *m*/*z *11,660**			**(*n*,*m*) Candidate formulas for *m*/*z *11,993**		
**Formula**	**Mass**	**Deviation from 11,660 Da**	**Formula**	**Mass**	**Deviation from 11,993 Da**
(35, 35)	11696.3	+36.3	(36, 36)	12030.5	+37.5
(36, 33)	11618.8	−41.2	(37, 34)	11952.9	−40.1
(36, 34)	11756.0	+90.0	(37, 35)	12090.2	+97.2
(37, 31)	11541.3	−118.7	(38, 32)	11875.5	−117.5
(37, 32)	11678.5	+18.5	(38, 33)	12012.7	+19.7
(38, 30)	11601.1	−58.9	(39, 31)	11935.3	−57.7
(38, 31)	11738.3	+78.3	(39, 32)	12072.4	+79.4
**(39, 29)**	**11660.8**	**−0.8**	(40, 29)	11875.7	−117.3
(39, 30)	11798.0	+138	**(40, 30)**	**11994.9**	**+1.9**
(40, 27)	11583.4	−76.6	(41, 28)	11917.5	−75.5
(40, 28)	11720.6	+60.6	(41, 29)	12054.7	+61.7
(41, 26)	11643.1	−16.9	(42, 27)	11977.2	−15.8
(41, 27)	11780.3	+120.3	(42, 28)	12114.4	+121.4
(42, 24)	11565.6	−94.4	(43, 25)	11899.7	−93.3
(42, 25)	11702.8	+42.8	(43, 26)	12036.9	+43.9
(43, 23)	11625.4	−34.6	(44, 24)	11959.5	−33.5
(43, 24)	11762.6	+102.3	(44, 25)	12096.7	+103.7
(44, 21)	11547.9	−112.1	(45, 22)	11882.0	−111
(44, 22)	11685.1	+25.1	(45, 23)	12019.2	+26.2
(45, 20)	11607.7	−52.3	(46, 21)	11941.9	−51.1
(45, 21)	11744.9	+84.9	(46, 22)	12078.9	+85.9

### Insight into the size-controlled synthesis of Au_39/40_ nanoclusters

The attainment of two new nanoclusters, Au_39_(SC_2_H_4_Ph)_29_ and Au_40_(SC_2_H_4_Ph)_30_, demonstrates the feasibility of controlling cluster size via controlled reduction. Herein, we discuss some insights obtained regarding the synthetic processes of Au_20_, Au_24_, and Au_39/40_. These four cluster species belong to a new series, as Au_20_ and Au_24_ are formed concurrently, albeit in small amounts, in the synthesis of Au_39/40_ nanoclusters. However, by controlling the reaction process, one may selectively produce Au_20_, Au_24_, or Au_39/40_. When using Au(III) as the starting material for nanocluster synthesis, two primary stages include (a) the reduction of Au(III) to Au(I) by HSR and (b) the conversion of Au(I) to Au_*n*_(SR)_*m*_ clusters by reduction with NaBH_4_. Factors that are important for Au_*n*_ size control with *n* = 20, 24, 39 and 40 include (a) the stirring speed of the reaction mixture, (b) the addition speed of NaBH_4_, and (c) the amount of NaBH_4_ added (*vide infra*).

### Stirring speed and effect on cluster size

We found the stirring speed is quite important for controlling the size and monodispersity of gold clusters. As listed in Table 2, during stage I, the stationary condition favors the formation of Au_20_(SR)_16_ nanoclusters, while slow stirring (50 to 100 rpm) favors the formation of Au_24_(SR)_20_, Au_39_(SR)_29_, and Au_40_(SR)_30_.

**Table 2:** **The respective conditions for the syntheses of Au**_**20**_**, Au**_**24,**_**and mixed Au**_**39/40**_

	**Cluster size**	**Stage I**	**Stage II**	**Amount of NaBH**_**4**_**(concentration, 0.44 M) per mole of gold and speed of addition**	**Aging time**
		**Stirring speed at the stage of Au(III) reduction to Au(I) by 3X HSC**_**2**_**H**_**4**_**Ph**	**Stirring speed at the stage of Au(I) reduction to clusters by NaBH**_**4**_		
1	Au_20_	Stationary	Approximately 50 rpm	1 mL (1×), dropwise added over a 30-min period	Approximately 6 hrs
2	Au_24_	50 to 100 rpm	Approximately 100 rpm	1 mL (1×), dropwise added over a 15-min period	Overnight
3	Au_39/40_	50 to 100 rpm	Approximately 400 rpm	1 mL (1×), dropwise added over a 15-min period	Overnight
4	Au_25_	50 to 100 rpm	50 to 400 rpm	5 mL (5×), Dropwise added over a 50-min period	Overnight

During stage II (the reduction of Au(I) by NaBH_4_), the stirring speed is even critical for the selective formation of Au_20_, Au_24_, or Au_39/40_. A slow stirring (approximately 50 rpm) during stage II favors the formation of Au_20_(SC_2_H_4_Ph)_16_, while a slightly higher speed of stirring (approximately 100 rpm) favored the formation of Au_24_(SC_2_H_4_Ph)_20_, and with further increased speed to approximately 400 rpm, we obtained new clusters of Au_39_(SC_2_H_4_Ph)_29_ and Au_40_(SC_2_H_4_Ph)_30_. This controlled reaction process for tuning cluster size is quite interesting. We believe that the aggregated Au(I)SR species are broken into small fragments upon reduction or partial reduction by NaBH_4_. Different stirring speed for stage II would influence the kinetics of the reduction reaction of Au(I)SR, and the different stirring speeds also give rise to different sheering forces that would break polymeric Au(I)SR into different sized fragments; such different sized fragments seem to subsequently grow into different sized clusters based upon our results.

Regarding the aggregated Au(I)SC_2_H_4_Ph species in the solution, structural characterization (e.g., by NMR or X-ray diffraction) is still difficult to carry out as the Au(I) intermediate species is poorly soluble in common solvents. Thermal gravimetric analysis determined the composition of Au(I):SR to be 1:1, but the aggregation degree (e.g., how many repeat units in [Au(I)SC_2_H_4_Ph]_*x*_) and what structures [Au(I)SC_2_H_4_Ph]_*x*_ may adopt are all unknown yet. Possible structures of [Au(I)SC_2_H_4_Ph]_*x*_ include linear chains, ring [53-57], or lamellar structures. The characterization of Au(I)SR still need major efforts in future work.

### Dropwise addition speed of NaBH_4_ and effect on cluster size

In addition to the stirring speed during stage II, the addition speed of NaBH_4_ (aqueous solution) to reduce Au(I) into clusters also plays an important role in controlling the final cluster size. We have tested that, in the synthesis of Au_20_(SC_2_H_4_Ph)_16_, if the initial drops of NaBH_4_ solution (0.44 mol/L, 1 mL) were added *rapidly*, the light yellow solution of Au(I) aggregates would rapidly become brown or deep black, and the product would contain more Au_24_ and Au_39/40_ clusters, instead of the predominant Au_20_ as the case of very slow addition of NaBH_4_ (over a period of approximately 30 min) (entry 1, Table 2). After optimization, we found that adding NaBH_4_ (0.44 mol/L, 1 mL, same concentration and amount as the Au_20_ synthesis) over a period of approximately 15 min gave rise to predominant Au_24_ (under approximately 100 rpm stirring condition) or Au_39/40_ (under approximately 400 rpm stirring condition); see entries 2 and 3 of Table 2. Thus, controlled reduction of Au(I) is very important for size selective formation of Au_20_, Au_24_, or Au_39/40_. The selective formation of Au_39/40_ over Au_24_ - which differ only in the stirring speed during stage II (i.e., 400 vs 100 rpm, Table 2) - should be due to the different reaction kinetics in the reduction process of Au(I) species into clusters. In a recent synthetical work on gold/phosphine nanocluster synthesis, Pettibone and Hudgens identified a growth-etching cyclic process that occurs around the most stable cluster species to form monodisperse product [4,58,59]. This size selective growth mechanism provides important information on the gold/phosphine system. The reaction kinetics of the gold/thiol system, however, still needs significant input in order to unravel the details of the kinetic process. Mass spectrometric monitoring of the reaction intermediates would provide valuable information and should be pursued in future work.

### Degree of reduction of Au(I) and effect on cluster size

With respect to the growth of Au_20_, Au_24_, and Au_39/40_ nanoclusters, an interesting question arises naturally: why the ubiquitous Au_25_ nanocluster is not formed under these conditions (entries 1 to 3, Table 2). The synthesis of Au_25_(SC_2_H_4_Ph)_18_ is typically done under experimental conditions of fast stirring and rapid reduction of Au(I) with large excess of NaBH_4_ (approximately 10 equivalents (eq) of NaBH_4_ per mole of gold). An important difference lies in that the syntheses of Au_20_, Au_24_, and Au_39/40_ clusters all involve *1 eq* of NaBH_4_ per gold, i.e., 1 mL of NaBH_4_ solution (0.44 mol/L), Table 2. This implies that the degree of reduction of Au(I) might affect the cluster size, and the formation of Au_25_ clusters might necessitate over reduction of Au(I) with a large excess of NaBH_4_.

To find out whether the reduction degree of Au(I) species affect the final cluster size, we adopt the same stirring conditions as the synthesis of Au_24_ and Au_39/40_ (see entry 4, Table 2), but we add more NaBH_4_ (e.g., 5 eq, rather than 1 eq for the synthesis of Au_24_ and Au_39/40_). The addition speed of NaBH_4_ solution is kept comparable to the syntheses of Au_24_ and Au_39/40_ clusters. Interestingly, dropwise addition of 5 eq of NaBH_4_ does result in selective formation of Au_25_, instead of Au_24_ or Au_39/40_, evidenced by its characteristic spectroscopic features (see Figure S2 in Additional file 1). Thus, the growth of Au_25_ nanoclusters does require a rich reductant (NaBH_4_), as opposed to lean NaBH_4_ for Au_20_, Au_24_, and Au_39/40_ synthesis. The fast stirring and rapid addition of NaBH_4_ seem not the key to the synthesis of Au_25_ nanoclusters.

## Conclusions

This work has demonstrated the effectiveness of controlled reduction for synthesizing different sized gold nanoclusters. Specifically, slow stirring and slow addition of 1 eq NaBH_4_/mol of gold are critical to effect the preferential growth of the series of Au_20_, Au_24_, and Au_39/40_ nanoclusters. In addition to the reaction kinetics, controlling the degree of reduction also leads to different sized nanoclusters, as demonstrated in the selective formation of Au_25_ over Au_*n*_ (*n* = 20, 24, 39/40). Future work is hoped to offer deeper mechanistic understanding of the Au(I) formation and the Au(I) reduction process by NaBH_4_. Mechanistic understanding of the cluster growth process will eventually permit high yielding synthesis of a full series of monodisperse gold nanoclusters.

## Abbreviations

NaBH_4_: sodium borohydride; TOAB: tetraoctylammonium bromide.

## Competing interests

The authors declare that they have no competing interests.

## Authors’ contributions

XM and ZL participated in all the studies and in writing this paper. MZ and RJ supervised in the concept of the study and participated in its design and in the revision of the manuscript. All authors read and approved the final manuscript.

## Authors’ information

XM is presently working at Anhui University (China). He received his PhD in Chemistry from the University of Science and Technology of China in 2007. His research interest focuses on chemosensors. ZL is a graduate student in the Zhu group at Anhui University (China). He obtained his BS in Chemistry (2010) from Anhui University. His research interest is noble metal nanoparticles. MZ presently works at Anhui University (China). He received his PhD in Chemistry from the University of Science and Technology of China in 2000. Before joining the Jin group as a postdoctoral researcher in February 2007, he worked at the University of Science and Technology of China. His research interests focus on photoinduced electron transfer, sensors, and nanomaterials. RJ received his BS in Chemical Physics from the University of Science and Technology of China (Hefei, China) in 1995, MS in Physical Chemistry/Catalysis from Dalian Institute of Chemical Physics (Dalian, China) in 1998, and PhD in Chemistry from Northwestern University (Evanston, Illinois) in 2003. After 3 years of postdoctoral research at the University of Chicago (Illinois), he joined the Chemistry faculty of Carnegie Mellon University in September 2006. His current research interests focus on atomically precise noble metal nanoparticles, evolution of their structure, electronic and optical properties, and utilizing these well-defined nanoparticles in catalysis, optics, sensing, and so forth.

## Supplementary Material

Additional file 1 **Mass spectrum in the range of *m*/*z *2,000 to 8,000 and comparison of the UV-vis spectrum.** Figure S1 shows the ESI mass spectrum in the range of *m*/*z* 2,000 to 8,000. Residual Au_24_(SC_2_H_4_Ph)_20_ clusters (in mono-Cs adducts) were observed at *m*/*z* 7603. The low mass portion contains all (CsAc)*n*Cs + signals with equal spacing of 191.9 (=CsAc), for example, the *m*/*z* 2,051.99 is assigned to (CsAc)_10_Cs^+^. Figure S2 shows the comparison of the UV-vis spectrum of the crude products (profile a) and of the purified Au_25_(SC_2_H_4_Ph)_18_ nanoclusters (profile b). Click here for file
