# Supplementary material for: Controlled reduction for size selective synthesis of thiolate-protected gold nanoclusters Aun(n = 20, 24, 39, 40)
Source: Nanoscale Res Lett. 2012 May 30;7(1):277. doi: 10.1186/1556-276X-7-277 (PMC3503687; doi:10.1186/1556-276X-7-277)
Supplement: Additional file 1 — Mass spectrum in the range of m/z 2,000 to 8,000 and comparison of the UV-vis spectrum. Figure S1 shows the ESI mass spectrum in the range of m/z 2,000 to 8,000. Residual Au24(SC2H4Ph)20 clusters (in mono-Cs adducts) were observed at m/z 7603. The low mass portion contains all (CsAc)nCs + signals with equal spacing of 191.9 (=CsAc), for example, the m/z 2,051.99 is assigned to (CsAc)10Cs+. Figure S2 shows the comparison of the UV-vis spectrum of the crude products (profile a) and of the purified Au25(SC2H4Ph)18 nanoclusters (profile b). [file 1556-276X-7-277-S1.pdf]

Supporting Information:

Controlled Reduction for Size Selective Synthesis of Thiolate-Protected Gold Nanoclusters Au_n (n=20, 24, 39, 40)

Xiangming Meng,[1] Zhao Liu,[1] Manzhou Zhu,*[1] and Rongchao Jin*[2]

[1]Department of Chemistry, Anhui University, Hefei, Anhui, 230026, P. R. China.

[2]Department of Chemistry, Carnegie Mellon University, Pittsburgh, Pennsylvania, 15213, USA.

1. Synthesis of $Au_{25}(SC_2H_4Ph)_{18}$ via slow reduction

$HAuCl_4{\cdot}3H_2O$ (0.1612 g, 0.41 mmol, dissolved in 5 mL Nanopure water) and tetraoctylammoniun bromide (TOAB, 0.2596g, 0.47 mmol, dissolved in 10 mL toluene) were combined in a 25 mL tri-neck round bottom flask. The solution was vigorously stirred (~1100 rpm) with a magnetic stir bar to facilitate phase transfer of Au (III) salt into the toluene phase. After ~15 min, phase transfer was completed, leaving a clear aqueous phase at the bottom of the flask; the aqueous was then removed using a 10 mL syringe. The toluene solution of Au (III) was cooled down to 0 °C in an ice bath over a period of 30 min under magnetic stirring. After stirring was reduced to a very low speed (~50 rpm), 2-phenylethanethiol (0.20 mL, ~3 equivalents of the moles of gold) was added. The solution was kept stirring after thiol addition, during which the solution color slowly changed from deep red to faint yellow, then gradually phased out and eventually became clear over a ~1 h period. After the solution turns to clear, an aqueous solution of $NaBH_4$ (0.0867g, 2.3 mmol, dissolved in 5 mL Nanopure water) was added *dropwise* to the solution within *50 minutes*. It is noted that during the addition of the $NaBH_4$, the stirring speed was kept very slow. After about 15 h, the crude product showed distinct absorption bands at 400, 450, 670 nm, which are characteristic absorption bands of $Au_{25}(SC_2H_4Ph)_{18}$ clusters (see Figure S1).

Post-synthetic treatment of the crude products: the aqueous layer at the bottom of the flask was removed using a syringe, and the organic phase was dried by rotary evaporation at room temperature. Ethanol (~40 mL) was added to precipitate the Au clusters. The precipitate was collected and redissolved in toluene and was precipitated again with ethanol. This dissolving-precipitation process was repeated twice. Very pure $Au_{25}(SCH_2CH_2Ph)_{18}^{-}TOA^{+}$ nanoclusters were obtained by extraction with acetonitrile.

2. Supporting Figures

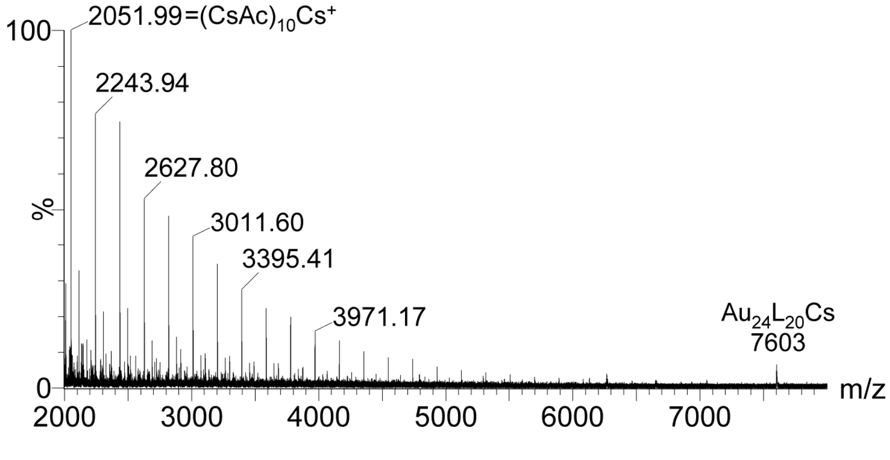

Figure S1. ESI mass spectrum in the range of m/z 2000-8000. Residual $Au_{24}(SC_2H_4Ph)_{20}$ clusters (in mono-Cs adducts) were observed at m/z 7603. The low mass portion contains all $(CsAc)_nCs^+$ signals with equal spacing of 191.9 (=CsAc), for example, the m/z 2051.99 is assigned to $(CsAc)_{10}Cs^+$.

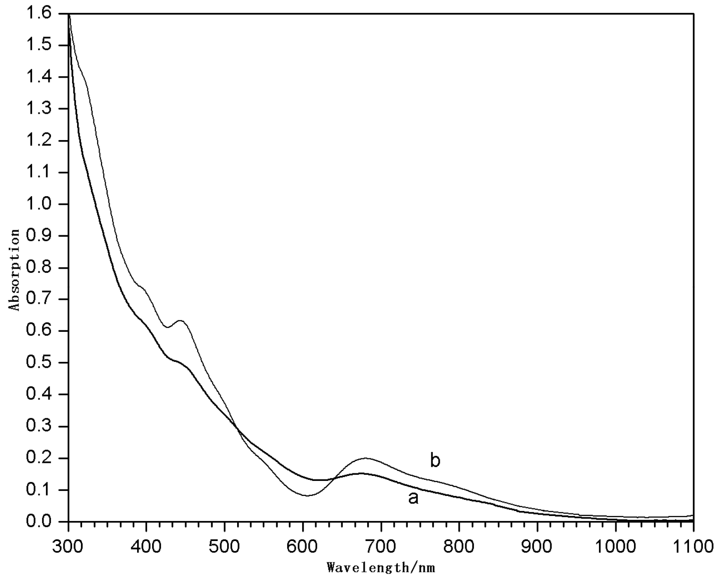

Figure S2. Comparison of the UV-Vis spectrum of the crude products (profile **a**) and of the purified $Au_{25}(SC_2H_4Ph)_{18}$ nanoclusters (profile **b**).
